# Fast peptide bond formation and release by the ribosomal large subunit

**DOI:** 10.1016/j.jbc.2025.110336

**Published:** 2025-06-03

**Authors:** Letian Bao, Anthony C. Forster

**Affiliations:** Department of Cell and Molecular Biology, Uppsala University, Uppsala, Sweden

**Keywords:** translation, ribosome, peptidyl transferase center, peptidyl release, puromycin

## Abstract

Peptide bond formation and peptidyl release are catalyzed at the peptidyl transferase center of the 50S subunit of the 70S ribosome. Proposed catalytic mechanisms at the peptidyl transferase center are based on structures of model substrates bound to the 50S and the 70S. Yet, peptidyl transfer and release reactions catalyzed by the 50S are slower by >3 orders of magnitude than those of the 70S. Here, we obtained a near-physiological rate of peptide bond formation with puromycin catalyzed by the 50S in 33% methanol at 37 °C, and fast rates were even attained in aqueous solution using 20% PEG. Interestingly, methanol, not PEG, accelerated the reaction by stimulating substrate binding just to the 50S P site. In addition, we obtained fast peptidyl release model reactions catalyzed by tRNA^Phe^ or Cytosine-Cytosine-Adenine (CCA) trinucleotide on the 50S in 30% acetone. However, PEG did not enable the release reaction, suggesting different mechanisms for release and peptide bond formation. The now-comparable peptidyl transfer rates of the 50S and 70S under aqueous conditions strengthen mechanistic proposals, give credence to hypothetical progenitor ribosomes before evolution of the 30S and will aid mechanistic investigations with model substrates or ancestral subsets of the ribosome.

The ribosome is a ribonucleoprotein complex central in protein synthesis. In bacteria, the 70S ribosome consists of a 30S small subunit and a 50S large subunit. The two primary reactions in translation, peptide bond formation and peptidyl release (hydrolysis), both occur at the peptidyl transferase center (PTC) of the 23S rRNA in the 50S. Although multiple other macromolecules are required in cells for peptidyl transfer, it was simplified *in vitro* to a “fragment reaction” between a 3′-terminal fragment of fMet-tRNA^fMet^ (or the whole fMet-tRNA^fMet^) and puromycin (Pmn) antibiotic catalyzed by just the 50S (Fig. S1) at 0 °C in 33% ethanol (1) or, more effectively, methanol (2). However, the physiological relevance of this widely used model reaction has been questioned (*e.g.* (3)) based on its extremely slow rate of ∼0.001/s for the fragment and 0.02/s for fMet-tRNA^fMet^ (2) and the need for organic solvent. While organic solvent was dispensable in fragment reactions with full-length fMet-tRNA^fMet^, the fastest Pmn reaction rate was only 0.008/s even at 37 °C (4). These rates are three to four orders of magnitude slower than ∼45/s for Pmn on mRNA-programmed 70S ribosomes with peptidyl tRNA (5), ∼160/s with aminoacyl-tRNA (6), or ∼20 amino acids/s *in vivo* (7). The low rates of the bacterial fragment reactions are apparently not due to incorrectly folded PTC because the aqueous PTC structure is identical in 50S and 70S crystals (8). This suggested to us the possibility of achieving faster fragment reactions by optimizing the conditions.

Translation termination requires a protein release factor (RF) to recognize the stop codon and hydrolyze the peptide from peptidyl-tRNA. However, *in vitro*, efficient hydrolysis of fMet-tRNA^fMet^ bound to AUG and the 70S was achieved using unacylated tRNA or CCA at the A site in 30% acetone (9) (see below Fig. 4*A*, left). Such RF-free release requires acetone rather than an alcohol because alcohols are sufficiently nucleophilic to switch the reaction from hydrolysis to ester formation. The only reported peptidyl release on 50S alone was by ribosomal protein L2 in aqueous solution (10), but it was ∼five orders of magnitude slower than RF-catalyzed release on 70S *in vitro* (Table 1, right). Nevertheless, we found that RF-free release on the 70S is A-site-codon-independent (11), suggesting the possibility of achieving mRNA-free, 30S-free, fast release. Here, motivated by the utility of simplified efficient ribosomal reactions, we aimed to accelerate dramatically 30S-free 50S reactions.

**Figure 1:**
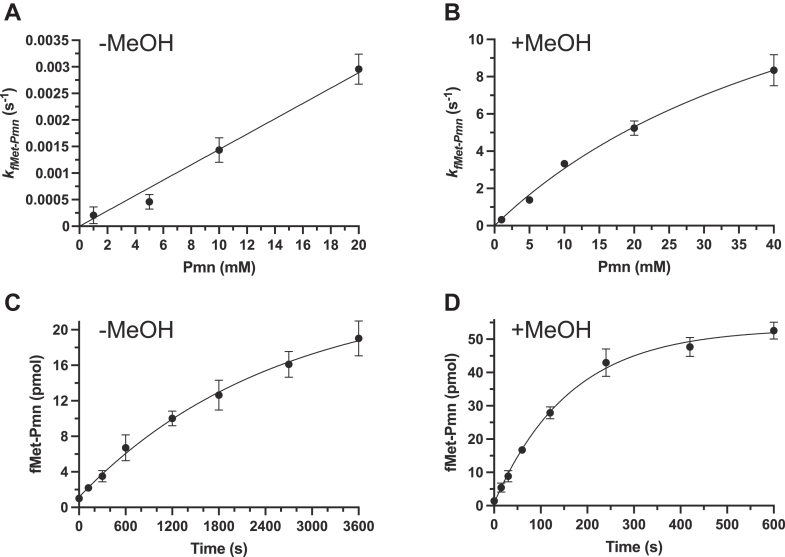
**Fragment reactions on 50S at 37 °C.** Single-round turnover rate dependence on Pmn concentration in aqueous (*A*) and 33% methanol (*B*) solution calculated from Fig. S2. Time courses of multiround turnover reactions in aqueous (*C*) and 33% methanol (*D*) solutions with 20 mM Pmn, 190 pmol fMet-tRNA^fMet^ and 9.5 pmol 50S. Error bars are SDs; n ≥ 3 technical replicates. MeOH, methanol; Pmn, puromycin.

**Figure 2:**
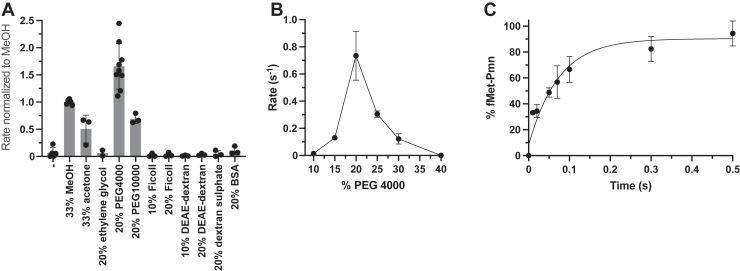
**Fragment reactions on 50S in PEG 4000.***A,* reaction rates of Pmn (1 mM) in different solutes normalized to the methanol rate calculated from Fig. S5. *B,* effect of PEG 4000 concentration on reaction with Pmn (1 mM). *C,* time course of fast Pmn reactions in 20% PEG 4000 with 40 mM Pmn. Error bars are SDs; n ≥ 3 technical replicates. Pmn, puromycin.

**Figure 3:**
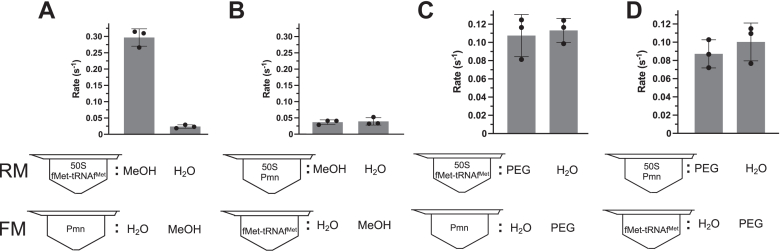
**Variation of preincubations.** Fragment reactions with 50S preincubated with P- or A-site (2 mM Pmn) substrates in 33% methanol (*A* and *B*) or 30% PEG 4000 (*C* and *D*). Note that for these experiments, these concentrations were not the final ones as they became diluted 2x in the fragment reactions. The resulting final PEG or MeOH concentrations were thus lower than optimal, a necessity due to their absence from one of the premixes. However, high stimulations were still observed. Error bars are SDs; n = 3 technical replicates. Pmn, puromycin.

**Figure 4:**
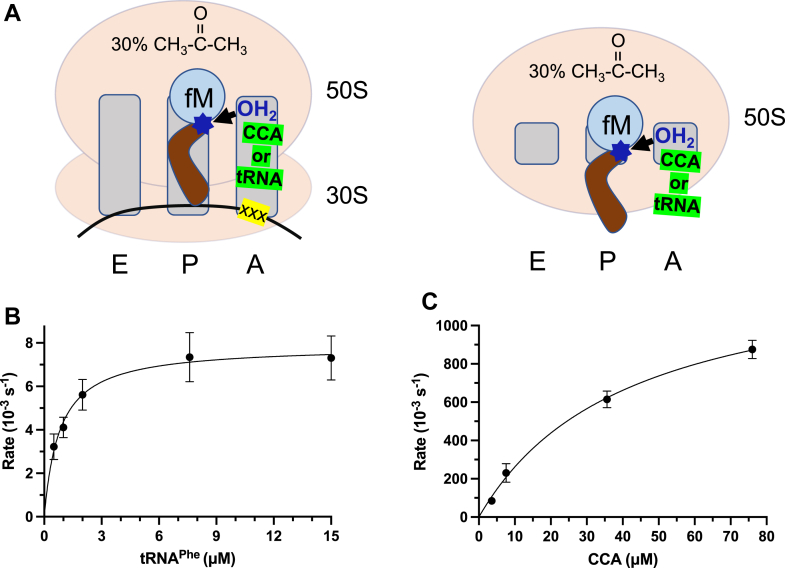
**Release-factor–independent peptidyl release on 50S.***A,* illustration of release-factor**–**independent release in the presence of 30% acetone on 70S (*left panel*; (9)) or 50S (*right panel*; this work). *B* and *C,* release rates from 50S plotted against concentrations of tRNA^Phe^ and CCA calculated from Fig. S9. Rates without tRNA^Phe^ and CCA have been subtracted. Error bars are SDs; n = 3 technical replicates.

**Table 1:** Maximal rates of 50S- and 70S-catalyzed peptidyl transfer (left) and release (right) from this study compared with literature values

Solution	Peptidyl transfer to fMet-tRNA^fMet^			Peptidyl release from fMet-tRNA^fMet^				
	50S + Pmn		70S + Pmn	50S			70S	
		Rate	Rate		Cofactor	Rate	Cofactor	Rate
Aqueous		0.008/s (4)	0.8/s (5); 45/s with fMANMFA-tRNA^Ala^		RF1	0	RF1	12.7/s (30)
					rp L2	0.0002/s (10)		
30% Organic solvent	MeOH	8.7/s		Acetone	RF1	0		
					tRNA^Phe^	0.0081/s		
					CCA	0.9/s		
20% PEG		13.6/s			tRNA^Phe^, CCA or RF1	0		

MeOH, methanol; Pmn, puromycin; RF, release factor.

## Results

### Peptide bond formation by the 50S subunit at a physiological rate

To optimize fragment reactions with fMet-tRNA^fMet^, we began by purifying 50S subunits (see Experimental procedures) and repeating the published (4) aqueous 37 °C reaction conditions with increasing Pmn concentrations (Fig. S2*A*). Our fastest measured single-round turnover rate of 0.003/s (Fig. 1*A* was comparable with the fastest rate published (Table 1, left). Although Pmn was not saturating in terms of rate, its solubility limit prevented higher concentrations. However, since methanol increases the solubility of Pmn and has been proposed to enhance P-site substrate binding in the fragment reaction (12), we shifted the solvent for the 37 °C reactions to 33% methanol (Fig. S2*B*). Historically, this may not have been attempted because fragment reactions with alcoholic conditions were performed on ice (13) probably due to the fear of substantial methanolysis side products at higher temperatures. But extraction into ethyl acetate of our 37 °C methanolic reaction products showed that methanolysis was only minor compared with fMet-Pmn formation (Fig. S3). So, we concluded that our new warm methanolic conditions accelerated peptide bond formation in the fragment reaction by a remarkable three orders of magnitude to 8.7/s (Fig. 1*B*), comparable with the average physiological protein synthesis rate of ∼20 amino acids/s (7).

As rates for single *versus* multiple turnovers have not been distinguished in fragment reactions, we next added fMet-tRNA^fMet^ at 20x excess of the 50S in our warm aqueous and methanol reactions (Fig. 1, *C* and *D*). Multiround turnover was faster with methanol (0.0068/s *versus* 0.0004/s) but much slower than single turnover, presumably due to the extra steps of tRNA^fMet^ dissociation and new fMet-tRNA^fMet^ binding. The faster turnover reaction (Fig. 1*D*) saturated well before converting all substrate to products. This was partially due to fMet-tRNA^fMet^ hydrolysis (Fig. S4*A*) as subsequent addition of fMet-tRNA^fMet^, but not 50S, enabled further rapid reaction (Fig. S4*B*). Rate limitation in the linear phase was independent of Pmn concentration (0.0068/s for 20 mM in Fig. 1*D*; 0.0064/s for 1 mM in Fig. S4*B*).

### PEG can substitute for methanol to enable assays that are more physiological

Although addition of methanol (or acetone; Fig. 2*A*) at physiological temperature enabled physiological reaction rates with the 50S “enzyme,” an organic solvent environment is unphysiological and not used in protein enzymatic reactions. Therefore, we wondered whether other solutes could substitute for organic solvent. Different solutes were tested in the linear portion of the time courses (Fig. S5) and the slopes normalized to methanol (Fig. 2*A*). Surprisingly, despite a long history of using organic solvents as stimulants, we found that aqueous 20% PEG 4000 massively accelerated the fragment reaction. Having been motivated to try it because of its well-known acceleration of biochemical reactions by molecular crowding, we thus tried other crowding reagents. Unexpectedly, they failed to stimulate, implying that simple crowding *per se* was not the reason for stimulation by PEG. PEG 10000 stimulated only half as much as PEG 4000, and the concentration of PEG 4000 exhibited a sharp optimum (Figs. S6 and 2*B*). PEG 4000 enabled an even faster rate than methanol, giving 13.6 ± 2.3/s with 40 mM Pmn (Fig. 2*C*; Table 1 left), but at a more physiological condition. However, high concentrations of PEG and 50S caused 50S precipitation (see Experimental procedures), and reducing the Mg^2+^ concentration from the standard 60 mM to 5 mM reduced the rates of the PEG and methanol reactions by more than an order of magnitude (not shown).

A unique advantage of our optimized 50S kinetic assay is the ability to directly analyze the effect of alterations in the large and complex 50S to define a minimal ribosome or key 50S residues without possible compensation by binding to the 30S. For example, our published fragment reactions with 50S from strains with KOs of rRNA modification enzymes were performed as end-point assays under highly unphysiological conditions (14, 15). We now compared the 50S subunits from the *ΔrlmE* strain (which shows 2–4-fold growth deficits compared to WT) and *ΔrluC/ΔrlmKL/ΔrlmN/ΔrlmM/ΔrluE* (abbreviated *ΔCKLNMuE*; note that rlmE is not deleted, which displays comparable growth at 37 °C but slower growth at 20 °C) with WT. These rRNA modifications cluster in the 3D structure of the PTC (rendered in Figure 2, Figure 3*G* in (14)). All of our optimized fragment kinetics assays, even under the most physiological conditions with PEG at 37 °C, revealed significant deficits for the KOs compared with WT (Fig. S7). This shows how our new assays can detect deficits that were not evident from fragment end-point assays or 70S dipeptide kinetics (15).

### Different mechanisms of acceleration by methanol and PEG

It was proposed that methanol stimulates the fragment reaction by enhancing substrate binding at the P site (12). To investigate this possibility, we took advantage of our preincubation step of the 50S. This step was done routinely because it is necessary in 70S reactions to achieve fast burst rates (16). We wondered whether the 50S reactions also benefited from preincubation and, if so, with what: methanol, P-site substrate, A-site substrate or some combination thereof? Indeed, a 5-min preincubation was necessary for fast fragment reactions (not shown), and this preincubation required a certain subset of components in the same tube: 50S, P-site substrate and methanol (Fig. 3, *A* and *B*, where preincubation of 50S, A-site substrate and methanol did not stimulate.) This supports a role for methanol in binding substrate to the P site, not the A site.

In the case of PEG, although molecular crowding *per se* did not appear to be the mechanism of acceleration (Fig. 2*A*), we nevertheless wondered if PEG, like methanol, also stimulated substrate binding to the P site. Unexpectedly, the same type of combination experiments now performed with PEG gave very different results from those with methanol (Fig. 3*C* and *D*): none of the components required preincubation with PEG for stimulation. So, either PEG does not enhance substrate binding or binding is no longer rate-limiting. Thus, the mechanisms of stimulation by PEG 4000 and methanol differ.

### Fast release by the 50S subunit

Having achieved physiological rates of peptide bond formation by the 50S, we aimed to do the same for the other translation reaction at the PTC, peptidyl-tRNA hydrolysis (release), which apparently occurs at a comparable rate (Table 1). However, this was an even bigger challenge because (i) release is co-catalyzed by an RF protein which recognizes the mRNA's stop codon on the 30S, (ii) the “fastest” release without RF on 50S alone was only 0.0002/s (requiring addition of ribosomal protein L2; Table 1), and (iii) alcohols could not be used because they favored ester formation over hydrolysis (9). We thus turned to the factor-free model of release from fMet-tRNA^fMet^ using unacylated tRNA or CCA catalyst at the A site in 30% acetone (9) (Fig. 4*A* left) to test if the 30S was dispensable in a 70S release reaction having a rate (0.3/s with CCA (11)) within two orders of magnitude of RF-catalyzed release (Table 1). Indeed, in the absence of the 30S and RF, fMet release rates up to 0.9/s were ultimately attainable (Table 1). This was achieved by first optimizing the concentration of the P-site substrate (Fig. S8) and then the A-site catalysts (Fig. S9). The K_M_ values of the tRNA^Phe^- and CCA-catalyzed release on 50S (Fig. S9; Fig. 4, *B* and *C*) were comparable to 70S (11) (for tRNA^Phe^: 0.87 *versus* 0.78 μM, respectively; for CCA: 36.8 *versus* 29.4 μM). However, the *k*_*cat*_ values on 50S were higher than on 70S with tRNA^Phe^ (0.0081 *versus* 0.0047/s) and much higher with CCA (1.3 *versus* 0.093/s). Interestingly, 20% PEG could not substitute for acetone in the tRNA^Phe^- or CCA-catalyzed reactions (Fig. S10), suggesting different mechanisms of peptide bond formation and release on the 50S. As expected, RF1 was unable to substitute for the model A-site catalysts in 30S-free release (Fig. S10). In summary, our new conditions using model A-site catalysts and acetone accelerated 30S-free release remarkably by more than three orders of magnitude to 0.9/s.

## Discussion

The reactions catalyzed by the 50S are thought to be mediated by the PTC RNA based on biochemical, structural and evolutionary evidence, although ribosomal proteins are required (17). Thus, literature on the effects of alcohols and PEGs on RNA structure and function is pertinent. Methanol can stabilize the tertiary interactions of RNA at the cost of destabilizing base pairing (18, 19, 20). In fragment reactions, the proposal that methanol enhances the binding of low molecular weight P-site substrates on 50S (12) is supported by Figure 3, *A* and *B*. This information thus suggests that methanol stabilizes the tertiary structure of the PTC–substrate complex rather than the two adjacent G-C pairs between substrate and P loop at the PTC (C74-G2252 and C75-G2251 (8)).

As PEG was the only crowding agent that stimulated (Fig. 2*A*), crowding *per se* did not appear to be the mechanism of stimulation. However, like methanol, PEG can stabilize the tertiary interactions of RNA. PEG accelerates and stabilizes RNA folding and favors more compact RNA structures by its well-known crowding effect (21, 22). PEG can stabilize yeast tRNAs (20) and longer range loop−loop interactions become more similar to those *in vivo* when buffers are supplemented with high-molecular-weight PEGs (23) such as PEG 4000. On the other hand, low-molecular-weight PEGs and ethylene glycol can destabilize RNA structure (23). Interestingly, procedures that precipitate the hairpin ribozyme with (24) or without PEG (25) stimulate catalysis. These phenomena fit with Figure 2*A* and associated 50S precipitation data (Experimental procedures). Furthermore, Figure 3, *C* and *D* showed no evidence of stimulation of substrate binding to enzyme by PEG. This information thus suggests that PEG stimulates peptidyl transfer by stabilizing the tertiary structure of the PTC. Regardless of the mechanism, it allows study of the reaction under more physiological conditions. Indeed, PEG was an additive used to produce the 50S and 70S crystal structures that are regarded as physiological by the field (8). However, PEG did not stimulate the other reaction at the PTC, release. This supports different mechanisms for release and peptide bond formation, as also suggested by a PTC mutagenesis experiment (A2602 mutation abolishes release, not peptidyl transfer (26)).

Our optimization of peptide transfer and release reactions on the 50S to near-physiological rates provides biochemical evidence, in addition to evidence from the 70S structures, which supports the relevance of mechanistic conclusions drawn from crystal structures of 50S complexes (8). It also opens up a new kinetic window for revisiting attempts to determine minimal components of the complex ribosome necessary for activity (17). As only the 50S catalyzes the chemical reactions of the ribosome, our finding that this catalysis is efficient means that it could also have been so early in evolution. Thus, coevolution of a proto-30S is an unnecessary pathway to a highly functional proto-50S. Such a proto-50S may have been selected for life's first syntheses of dipeptides and even short polypeptides in an untemplated manner from progenitor aminoacyl-tRNAs (27).

## Experimental procedures

### Materials

Tritium-labeled Met was purchased from PerkinElmer. All other chemicals and reagents were purchased from Sigma-Aldrich or Merck. f[^3^H]Met-tRNA^fMet^ was prepared as described (28). An active concentration of 60% compared with tRNA content (measured by A_260_) was determined by HPLC titration of product formation with 50S ribosomes (*e.g.* Fig. S2*C*). Preparation of the three chromosomal mutant strains *ΔrlmE, ΔrluC/ΔrlmE, and ΔrluC/ΔrlmKL/ΔrlmN/ΔrlmM/ΔrluE* (*ΔCKLNMuE*) in MG1655 has been detailed (14). MRE600 was used as “WT” for KO 50S fragment reaction experiments due to lower RNase I activity than MG1655 WT.

### Ribosomes and their subunits

70S ribosomes from *Escherichia coli* were purified by four ultracentrifugations as described (6) with two minor modifications: (i) the Mg(OAc)_2_ in both sucrose cushions was 10.5 mM instead of 10 and (ii) centrifugation through the second sucrose cushion was at 28,000 rpm for 18 h. 50S subunits were isolated from 70S ribosomes (similar to (29)) by diluting the 70S in overlay buffer containing 20 mM Tris–HCl (pH 7.5), 300 mM NH_4_Cl, 3 mM Mg(OAc)_2_, 0.5 mM EDTA, and 3 mM 2-mercaptoethanol. The diluted solution was then loaded onto a 10 to 35% sucrose gradient in overlay buffer and centrifuged at 32,000 rpm in a Ti-15 rotor at 4 °C for 9 h to yield separate 50S and 30S peaks (Fig. S11); only the upper two-thirds of the 50S peak was collected. The 50S was pelleted and resuspended as described for the 70S (6). Its concentration measured by A_260_ roughly matched its active concentration measured by the end time point of the fast phase of the fragment reaction. Given concerns that some or much of the activity measured in aqueous fragment reactions may be due to 70S assembled from 30S contaminating the 50S peak (4), we measured the initial rate of fMet-Pmn formation from 1 mM Pmn by the 70S in 20% PEG 4000. This rate was 0.28/s ± 0.2/s (data not shown), 2.6× slower than for the 50S (Fig. 2*B*). Taken together with the lack of a 16S rRNA band upon agarose gel electrophoresis of 50S extracted with phenol/chloroform, we conclude that 70S contamination, if any, was not responsible for our fast fragment reaction rates.

### Methanolysis reactions

Reactions (45 μl) contained 11.3 pmol 50S and 22.5 pmol f[^3^H]Met-tRNA^fMet^ in 50 mM Tris–HCl (pH 7.5), 400 mM KCl, 60 mM MgCl_2_. Pmn (1 mM), MeOH (final concentration 33%), or water were included (Fig. S3) for incubation on ice or at 37 °C for 20 min. Reactions were diluted in 450 μl 0.1 M imidazole (pH 6.0), mixed with 1.5 ml ethyl acetate, and the organic and aqueous phases were separated by centrifugation. A 500 μl portion of the organic phase was taken from the top, the middle portion was discarded and 250 μl of aqueous phase was taken from the bottom. The phases were mixed with 5 ml ProFlow G+ scintillation liquid for counting (Beckman Coulter LS 6500). After correcting for the different partial fractions analyzed, the ratio of products to reactants was calculated as [^3^H]organic/([^3^H]organic + [^3^H]aqueous).

### Peptidyl transferase reactions

Full-length f[^3^H]Met-tRNA^fMet^ (1 μM unless otherwise indicated) was used as the P-site substrate in a ribosome mix (RM, total 147 μl) containing 0.5 μM 50S subunit, 50 mM Tris–HCl (pH 7.5), 400 mM KCl, 60 mM MgCl_2_, and the solutes or solvents indicated (*e.g.* Fig. 2*A*). Upon addition of PEG 4000 or 10000, but not other crowding agents, to the 50S, gel-like milky precipitation of the 50S occurred. Despite this, very efficient substrate binding and reaction still occurred (Fig. 3, *C* and *D*; in control experiments, centrifugation pelleted essentially all A_260_ units and resuspension yielded highly active 50S). For kinetics in quench flow (Figs. 2*C*, S2*B* and S7), 2 μM f[^3^H]Met-tRNA^fMet^ and 1 μM 50S were used due to signal detection limitations. From the RM, 12 μl was withdrawn for the 0 time point, leaving 135 μl for the reaction. A factor mix (FM, 135 μl) contained the same buffer and solvent/solute composition but with Pmn at the concentrations indicated instead of f[^3^H]Met-tRNA^fMet^ and 50S. Both RM and FM were preincubated individually at 37 °C for 5 min (or on ice for 10 min for reactions on ice), followed by rapid mixing and quenching with 0.4 M final concentration of KOH at indicated time points manually or in a temperature-controlled quench-flow apparatus (RQF-3, KinTeck Corp.). The quenched reactions were incubated at 37 °C for 10 min for hydrolyzing the remaining f[^3^H]Met-tRNA^fMet^, followed by adding 17% final concentration of formic acid and centrifuging at 20,000*g* for 30 min at 4 °C. The supernatant was analyzed by C18 reversed-phase HPLC coupled with a β-RAM model 3 radioactivity detector (IN/US Systems). Separation of f[^3^H]Met and f[^3^H]Met-Pmn was achieved by elution with 50% methanol/50% H_2_O/0.1% trifluoroacetic acid for 20 min at 0.45 ml/min. The fraction of [^3^H] peptide out of the total [^3^H] signal at each time point was calculated and the data fitted to a single exponential function with Origin 7.5 (OriginLab Corp., http://www.originlab.com/ftp/dist/origin75sr7/Origin7.5SR7.exe).

### Variation of preincubations

The preparation of the RM and FM were identical to the fragment reactions except that (i) in the RM, 50S was preincubated either with f[^3^H]Met-tRNA^fMet^ or Pmn as indicated in the Figure 3, and (ii) the MeOH or PEG 4000 concentrations of Figure 3 were added to only RM or FM. The RM and FM were preincubated individually at 37 °C for 5 min. The subsequent reactions and sample analysis were the same as described in fragment reactions.

### Release reactions

The preparations of RM and FM (without Pmn), preincubation, and mixing were as for the fragment reaction except 100 mM MgCl_2_ and 0.2 μM 50S were used, and either tPhe or CCA were added at the concentrations shown in Figure 4, *B* and *C*. Release on the ribosome is almost two orders of magnitude faster by tPhe, and much faster still by CCA, than reactions omitting the 50S. Reactions were quenched with 17% formic acid and supernatants containing released f[^3^H]Met were separated from pellets containing the f[^3^H]Met-tRNA^fMet^ by centrifuging at 20,000*g* for 30 min at 4 °C. Pellets were dissolved in 120 μl 0.5 M KOH and incubated at 37 °C for 15 min. The radioactive supernatants and dissolved pellets were diluted in ProFlow G+ scintillation liquid for counting (Beckman Coulter LS 6500). The fraction of release was calculated as [^3^H] supernatant/([^3^H] supernatant + [^3^H] pellet).

## Data availability

The original data is available upon request.

## Supporting information

This article contains supporting information (31).

## Conflict of interest

The authors declare that they have no conflicts of interest with the contents of this article.
