# Supplementary material for: Fast peptide bond formation and release by the ribosomal large subunit
Source: J Biol Chem. 2025 Jun 3;301(7):110336. doi: 10.1016/j.jbc.2025.110336 (PMC12269836; doi:10.1016/j.jbc.2025.110336)
Supplement: Figures S1-S11 [file mmc1.docx]

***Communication***

**SUPPORTING INFORMATION**

**Fast peptide bond formation and release**

**by the ribosomal large subunit**

**Letian Bao and Anthony C. Forster^*^**

Department of Cell and Molecular Biology, Uppsala University, Husargatan 3, Box 596, Uppsala 75124, Sweden.

^*^To whom correspondence may be addressed. E-mail: [a.forster@icm.uu.se](mailto:a.forster@icm.uu.se)

Contents

**Additional reference**

Wilson DN, Nierhaus KH. The E-site story: the importance of maintaining two tRNAs on the ribosome during protein synthesis. *Cell Mol. Life Sci.* 2006, 63: 2725-37.

**Table 1 in original format**

**Supporting Figures 1-11**

**Graphical abstract**

**Table 1**. Maximal rates of 50S- and 70S-catalyzed peptidyl transfer (left) and release (right) from this study compared with literature values.

**Sup. Figure 1.** One of the “fragment reactions” catalyzed by the ribosomal 50S subunit alone in 33% alcohol. Chemical differences from the aminoacyl-tRNA reaction are in blue. Adapted from Wilson & Nierhaus (2006).

**Sup. Figure 2**. Time courses of reactions with different Pmn concentrations in aqueous (A) and 33% methanol (B) solutions at 37℃. For clarity, the data in (B) are plotted in two different scales. (C) HPLC traces showing early consumption of f[^3^H]Met-tRNA (red, region 1) and f[^3^H]Met-Pmn formation (green, region 2) over time from left to right with 1 mM Pmn in (B). The first of the two red peaks does not form product and increases slowly with storage, indicating radiolytic or oxidative decay. Error bars are standard deviations; n≥3 technical replicates.

**Sup. Figure 3**. Methanolysis product in reactions with Pmn (1 mM) at saturation (end) time points at 0℃ (blue) and 37℃ (red). The expected main distribution of 3H is fMet-tRNA^fMet^ (unreacted) and fMet (from spontaneous hydrolysis) in the aqueous phase, and fMet-Pmn and fMet-OCH_3_ (methanolysis product) in the organic phase. Error bars are standard deviations; n=3 technical replicates.

**Sup. Figure 4**. Substrate hydrolysis under multi-round turnover conditions. (A) Spontaneous hydrolysis of fMet-tRNA^fMet^ in aqueous (blue) and 30% methanol (red) ribosome-free solutions at 37℃. fMet-tRNA^fMet^ and fMet were separated by precipitation in 17% formic acid and centrifugation, and the supernatant was analyzed by HPLC. (B) Multi-round turnover reactions on 50S using 1mM Pmn with addition of extra fMet-tRNA^fMet^ (brown) or 50S (black) at 960 seconds. Error bars are standard deviations; n=3 technical replicates.

**Sup. Figure 5**. Determination of the relative rates of fragment reactions in different solutes at 1 mM Pmn. Slopes were calculated based on the linear increasing part of the fMet-Pmn formation time courses at 37℃. Error bars are standard deviations; n≥3 technical replicates.

**Sup. Figure 6**. Time courses of fragment reactions in different concentrations of PEG 4000 with 1 mM Pmn at 37℃. Error bars are standard deviations; n≥3 technical replicates.

**Sup. Figure 7**. Fragment reactions on 50S subunits isolated from strains with rRNA modification enzyme KOs using 10 mM Pmn in 33% methanol at 0℃ (A) and 37℃ (B, left panel) or with 40 mM Pmn and 20% PEG 4000 (B, right panel). Error bars are standard deviations; n≥3 technical replicates; calculated p values are based on one-tailed t-test.

**Sup. Figure 8**. Time courses (A) and their associated rates (B) at various fMet-tRNA^fMet^ concentrations of peptidyl release on 50S with 7.6 μM tPhe in 30% acetone at 37 ℃. Error bars are standard deviations; n=3 technical replicates.

**Sup. Figure 9**. Time courses of peptidyl release on 50S with different tRNA^Phe^ (A) and CCA (B) concentrations in 30% acetone at 37℃. For clarity, the data in (B) are plotted in two different scales. Error bars are standard deviations; n=3 technical replicates.

**Sup. Figure 10**. Peptidyl release on 50S with saturation concentration of different A-site catalysts in 30% acetone or 20% PEG 4000 for 1 min at 37℃. The left bar shows background hydrolysis. Error bars are standard deviations; n≥3 technical replicates.

**Sup. Figure 11.** Ribosome sucrose gradient profile for isolating 50S large ribosome subunits. The 70S was diluted with 3 mM Mg^2+^ to allow the separation of 50S and 30S and passed through a 10% to 35% sucrose gradient. The 50S peak was collected and concentrated by ultra-centrifuging.

**Graphical abstract**

The 50S subunit of the ribosome catalyzes peptide bond formation (shown) and release, but these reactions are extremely slow in the absence of the 30S subunit. Here, we achieve physiologically-relevant reaction rates on the 50S alone, e.g. by adding polyethylene glycol (PEG). Utility is demonstrated using rRNA modification enzyme knockouts.

----
